# Disseminated Varicella Zoster and Multiorgan Failure Requiring ECMO Support in a Patient With Evans Syndrome

**DOI:** 10.1155/crcc/2103304

**Published:** 2026-09-30

**Authors:** Rachel E. Gahagen, Riad Lutfi, Mara E. Nitu, Jatinder K. Dhami, John C. Christenson, Daniel T. Cater

**Affiliations:** ^1^ Division of Pediatric Critical Care Department of Pediatrics Northwestern University Feinberg School of Medicine Chicago Illinois USA northwestern.edu; ^2^ Division of Pediatric Critical Care Department of Pediatrics Indiana University School of Medicine Indianapolis Indiana USA iusb.edu; ^3^ Division of Pediatric Critical Care Department of Pediatrics Methodist Children′s Hospital San Antonio Texas USA; ^4^ Ryan White Center for Pediatric Infectious Diseases and Global Health Department of Pediatrics Indiana University School of Medicine Indianapolis Indiana USA iusb.edu

## Abstract

Visceral disseminated varicella‐zoster virus (VD‐VZV) infection is an uncommon but potentially life‐threatening manifestation of varicella infection that primarily affects immunocompromised patients. When hematogenous dissemination occurs, the risk of significant organ dysfunction and death increases considerably. The disease develops through systemic viral spread to visceral organs, resulting in widespread multisystem involvement. Patients with Evans syndrome may be at increased risk because of underlying immune dysregulation and the frequent need for immunosuppressive treatment. We report the case of a 5‐year‐old unvaccinated child with Evans syndrome who developed severe multiorgan failure secondary to VD‐VZV infection. The patient′s condition was refractory to medical management but was successfully supported with venoarterial extracorporeal membrane oxygenation (VA‐ECMO), without bleeding complications despite profound thrombocytopenia. A modified anticoagulation strategy and aggressive platelet support were used. The patient was successfully decannulated after 8 days of ECMO without major hemorrhagic complications and subsequently recovered renal and hepatic function. This case highlights the fulminant nature of VD‐VZV infection in an unvaccinated patient with Evans syndrome. It demonstrates that survival is possible with timely initiation of antiviral therapy and extracorporeal support.

## 1. Introduction

Varicella‐zoster virus (VZV), a member of the Orthoherpesviridae family, is a highly contagious human pathogen responsible for two clinically distinct syndromes: primary infection, or varicella (chickenpox), and herpes zoster, which results from reactivation of latent virus later in life (1). Primary varicella is generally a mild and self‐limited illness in otherwise healthy children. However, severe and disseminated disease has long been recognized in patients with impaired cellular immunity. Weller′s seminal description of varicella and herpes zoster emphasized the importance of host cellular immunity in controlling VZV and the markedly increased risk of severe disease among immunocompromised patients (1). Following inhalational exposure, VZV initially replicates in the upper respiratory tract and regional lymphoid tissue, followed by primary viremia and subsequent dissemination to reticuloendothelial tissues. A secondary viremic phase results in delivery of virus to the skin and other tissues, producing the characteristic vesicular exanthem. In immunocompetent hosts, coordinated innate and adaptive immune responses generally limit viral replication and terminate viremia. Impaired cellular immunity, however, can permit sustained viral replication and hematogenous dissemination, resulting in visceral disease (2).

Disseminated VZV infection is an uncommon but severe manifestation of VZV disease that primarily affects individuals with impaired immune function and can be associated with substantial morbidity and mortality. In a recent scoping review of 156 reported cases, the overall mortality was 25% and multiple visceral organs were involved in 46.1% of patients. The lungs were the most affected organs (56%), followed by the liver (44%), heart (16%), kidneys (11%), pancreas (11%), stomach (10%), and esophagus (6%). Acute kidney injury, hepatitis, acute liver failure, and septic shock or multiorgan dysfunction were associated with increased mortality. Although most reported patients were adults, 21 patients in this review were younger than 18 years, highlighting the limited pediatric experience with this uncommon complication (3). The diagnosis of disseminated VZV can be difficult because its presentation may resemble other infectious or inflammatory disorders. Rapid identification of the disease, early antiviral administration, and intensive supportive management are critical for improving survival. Although severe VZV pneumonia may necessitate invasive mechanical ventilation, extracorporeal membrane oxygenation (ECMO) has been infrequently reported and is typically considered only in cases of refractory respiratory or cardiopulmonary failure (4, 5).

Evans syndrome (ES) is an uncommon autoimmune hematologic disorder characterized by immune‐mediated destruction of multiple blood cell lines, most commonly autoimmune hemolytic anemia and immune thrombocytopenia, with occasional neutropenia. Patients may experience recurrent or persistent cytopenias, bleeding complications, transfusion requirements, and complications related to both immune dysregulation and immunosuppressive therapy. Pediatric ES is rare and is associated with significant morbidity and mortality (6). Immunosuppressive therapies used in affected patients may further increase susceptibility to severe opportunistic and viral infections.

In this report, we describe a critically ill, unvaccinated child with ES who developed disseminated VZV infection complicated by severe thrombocytopenia, circulatory shock, acute kidney injury, and refractory hypoxemic respiratory failure requiring venoarterial extracorporeal membrane oxygenation (VA‐ECMO). The case highlights the potential severity of disseminated VZV in children with underlying immune dysregulation and the potential role of extracorporeal support in patients with refractory cardiopulmonary failure.

## 2. Case Presentation

A 5‐year‐old unvaccinated female with a history of ES presented to the emergency department (ED) following known exposure to varicella within her mostly unvaccinated religious community. She had experienced several days of progressive fever, malaise, and decreased oral intake.

Prior to presentation, the patient was receiving treatment for ES with sirolimus and romiplostim for the 6 months preceding presentation, after steroids had been weaned. She was also receiving sulfamethoxazole and trimethoprim for *Pneumocystis jirovecii* pneumonia prophylaxis. Her last sirolimus dosing was 0.9 mg twice a day with the level in therapeutic range.

According to her family, she initially developed a diffuse pruritic rash beginning on her scalp and subsequently spreading throughout her body. She initially had only low‐grade fevers and otherwise remained in her usual state of health. One day prior to presentation, she became increasingly fatigued with worsening oral intake, although urine output remained adequate. On the day of admission, she became difficult to arouse, refused oral intake, and developed a fever with a maximum temperature of 38.8°C. Her family also noted facial and periorbital edema, along with increased work of breathing, prompting presentation to the ED.

On arrival, vital signs were notable for fever (38.5°C), tachycardia (heart rate 180–200 beats/min), hypoxemia (oxygen saturation 79% on room air), and hypotension (blood pressure 60/30 mmHg). Her weight was 19.6 kg. Initial arterial blood gas analysis demonstrated severe acidosis (pH 7.02, PaCO_2_ 58 mmHg, lactate 5 mmol/L). Serum creatinine on admission was 0.63 mg/dL (reference range, 0.3–0.7 mg/dL). Chest radiograph revealed diffuse bilateral ground‐glass opacities (Figure 1A). Because of worsening hypoxemia and altered mental status, the patient was intubated. She received isotonic fluid resuscitation and was started on epinephrine for hemodynamic support. Broad‐spectrum antibiotics and intravenous acyclovir were initiated for presumed septic shock and suspected disseminated varicella infection. Acyclovir was administered at a dose of 380 mg (approximately 20 mg/kg) intravenously every 8 h, with subsequent renal dose adjustment during dialysis. She was subsequently transferred to the pediatric intensive care unit (PICU).

**Figure 1:**
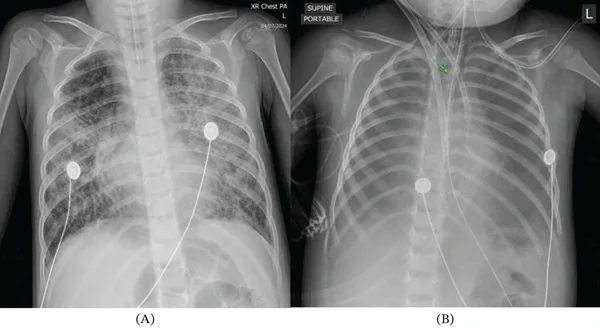
(A) Chest X‐ray at admission to the ICU. (B) Chest XR after ECMO cannulation.

Upon PICU admission, the patient remained persistently hypotensive and hypoxic despite escalation of care. Additional fluid resuscitation was administered, and vasoactive support was escalated with epinephrine, norepinephrine, and corticosteroids. Despite invasive mechanical ventilation, she developed refractory hypoxemia and was transitioned to high‐frequency oscillatory ventilation without significant improvement, with oxygen saturations persistently remaining in the 70s, with oxygenation index greater than 40.

Laboratory evaluation demonstrated marked leukocytosis (white blood cell count 43 × 10^9^/L) and severe thrombocytopenia (platelet count 10 × 10^9^/L). Given refractory hypoxemia, hemodynamic instability, and circulatory shock, the multidisciplinary team discussed candidacy for VA‐ECMO. Despite the substantial risk of bleeding in the setting of profound thrombocytopenia and disseminated viral infection, the decision was made, with family consent, to proceed with ECMO cannulation (Figure 1B).

Serum polymerase chain reaction (PCR) testing confirmed VZV infection. VZV PCR was a laboratory‐developed test performed on the QuantStudio 12K Flex platform (Thermo Fisher Scientific, Waltham, Massachusetts); the assay was developed and its performance characteristics determined by ARUP Laboratories (Salt Lake City, Utah), and testing was performed in a CLIA‐certified laboratory for clinical purposes. Respiratory viral panel testing was additionally positive for adenovirus, parainfluenza Type 2, and rhinovirus/enterovirus. The patient was treated with intravenous acyclovir, intravenous immunoglobulin (IVIG), and frequent platelet transfusions. IVIG was administered at a dose of 400 mg/kg as adjunctive therapy during her critical illness, with the goals of immunoglobulin replacement and potential modulation of the inflammatory response. The patient did not receive varicella‐zoster immune globulin (VZIG), as she presented with established disseminated VZV infection and clinical deterioration rather than during a postexposure prophylaxis window. Because of persistent severe thrombocytopenia, romiplostim was administered as adjunctive therapy. During ECMO support, platelet counts were maintained above 50 × 10^9^/L. A modified anticoagulation strategy was used, targeting reduced heparin intensity with an anti‐Xa goal of 0.3–0.5 IU/mL. Given her altered mental status and disseminated VZV infection, lumbar puncture was considered to evaluate for possible VZV encephalitis. However, persistent profound thrombocytopenia created a substantial bleeding risk. After multidisciplinary consideration of the risks and potential benefits, lumbar puncture was deferred.

The patient demonstrated gradual cardiopulmonary recovery while receiving VA‐ECMO and was successfully decannulated after 8 days of support without major hemorrhagic complications.

Cutaneous management of disseminated varicella was primarily supportive and focused on maintaining skin integrity and preventing secondary infection. Daily gentle cleansing with lukewarm water and mild nonirritating cleansers was performed, followed by careful pat drying. Pruritus was managed with topical calamine lotion and intermittent oral antihistamines as needed. Ruptured vesicles were cleansed and covered with nonadherent dressings when necessary. The patient was closely monitored throughout her hospitalization for signs of secondary bacterial infection (Figure 2).

**Figure 2:**
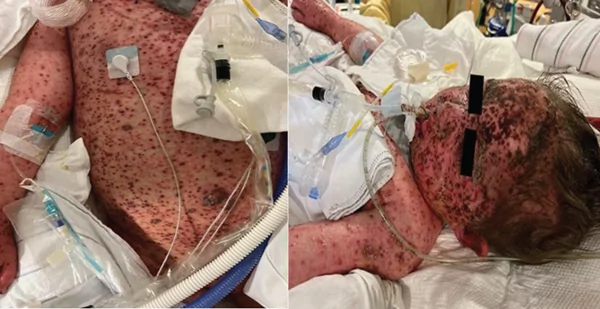
Diffuse cutaneous varicella‐zoster virus rash.

Her hospital course was further complicated by acute renal failure requiring temporary dialysis support; however, renal function recovered over the subsequent 2 weeks, and dialysis was discontinued. Serum creatinine was 0.87 mg/dL at ICU discharge and 0.21 mg/dL at hospital discharge. She later required tracheostomy and prolonged mechanical ventilation because of neuromuscular weakness but was successfully liberated from ventilatory support 4 weeks after her acute illness and no longer required supplemental oxygen. Following 2 weeks of inpatient rehabilitation, she was discharged home on valacyclovir prophylaxis after 7 weeks of hospital stay.

The patient′s subsequent course was complicated primarily by persistent hematologic morbidity related to ES. Approximately 2 months after hospital discharge, she was readmitted following a traumatic injury complicated by subdural hemorrhage. Persistent thrombocytopenia required frequent platelet transfusions; neurosurgical intervention was not required. Her renal and hepatic function remained recovered without persistent renal or hepatic complications. However, she continued to experience significant chronic morbidity and required close outpatient follow‐up, including follow‐up with palliative care.

Approximately 2 years after the initial admission, a do‐not‐resuscitate status was established. She was subsequently readmitted with pneumonia and ultimately died from hypoxemia after a decision to limit further escalation of medical therapy.

## 3. Discussion

Visceral disseminated VZV infection is an uncommon but potentially life‐threatening complication of varicella that occurs predominantly in patients with impaired cellular immunity (1, 2). Hematogenous dissemination can result in multisystem involvement, including pneumonitis, hepatitis, encephalitis, acute kidney injury, and cardiovascular collapse (2, 3). Diagnosis may be challenging because the clinical presentation can overlap with other infectious and inflammatory conditions; therefore, early recognition and initiation of intravenous acyclovir are critical (7). Although disseminated VZV is well described in immunocompromised patients, pediatric cases remain uncommon, and experience with the management of fulminant multisystem disease is limited (3).

ES is a rare autoimmune hematologic disorder characterized by immune‐mediated destruction of two or more blood cell lines, most commonly autoimmune hemolytic anemia and immune thrombocytopenia, with or without autoimmune neutropenia (6). Pediatric ES is associated with recurrent cytopenias, bleeding complications, and significant morbidity, whereas immune dysregulation and immunosuppressive therapy may increase susceptibility to severe infections (6). The association between ES and severe varicella has rarely been reported. Rahma et al. (8) described a 41‐year‐old woman with ES who developed hemorrhagic varicella with generalized hemorrhagic vesicles, mucosal bleeding, anemia, and thrombocytopenia and improved with antiviral and supportive therapy. Our patient developed substantially more severe disease, with disseminated VZV complicated by refractory hypoxemic respiratory failure, circulatory shock, acute kidney injury, encephalopathy, and multiorgan dysfunction. Thus, this case adds to the limited literature describing severe varicella in patients with ES and highlights the potential for fulminant visceral disease in children with underlying immune dysregulation.

Previously reported pediatric cases of disseminated varicella provide context for the present case. Kim et al. (9) described an immunocompromised child with acute lymphoblastic leukemia who developed disseminated varicella complicated by acute respiratory distress syndrome, disseminated intravascular coagulation, encephalopathy, and hepatitis. This child was treated with acyclovir, IVIG, and invasive mechanical ventilation and survived without need for extracorporeal support. Pandya et al. (10) described a case series of VZV infection in pediatric solid organ transplant recipients in the prevaccine era. Our patient differs in several respects: Her underlying condition was an autoimmune cytopenia versus malignancy or transplantation, her thrombocytopenia was profound at presentation, and she required VA‐ECMO support for combined refractory hypoxemia and shock rather than ventilatory support alone. We are unaware of a previously reported pediatric case of disseminated varicella in ES and supported with ECMO.

The need for VA‐ECMO presented a particular challenge because of profound thrombocytopenia, with an initial platelet count of 10 × 10^9^/L. Although ECMO has been reported in severe varicella infection, published experience is limited and has predominantly involved adults with varicella pneumonitis and refractory respiratory failure (4, 5). In our patient, both refractory hypoxemic respiratory failure and circulatory shock necessitated VA‐ECMO. Given the otherwise high risk of death, a multidisciplinary decision was made to proceed with extracorporeal support despite the bleeding risk. A modified anticoagulation strategy targeting an anti‐Xa level of 0.3–0.5 IU/mL was used, with platelet transfusions to maintain counts above 50 × 10^9^/L and continued romiplostim therapy. The patient was successfully decannulated after 8 days without major hemorrhagic complications. This experience demonstrates that profound thrombocytopenia, while presenting a substantial challenge, does not necessarily preclude extracorporeal support when the severity of cardiopulmonary failure warrants it.

Several aspects of this case also illustrate the diagnostic challenges associated with disseminated VZV. The patient had altered mental status concerning possible VZV encephalitis, but lumbar puncture was deferred because of the bleeding risk associated with profound thrombocytopenia. Similarly, detection of adenovirus, parainfluenza Type 2, and rhinovirus/enterovirus complicated interpretation of her respiratory failure, although the available clinical data did not allow these infections to be distinguished as independent contributors from disseminated VZV. Her acute kidney injury was likely multifactorial in the setting of shock, systemic inflammation, and potential medication‐related or VZV‐associated renal injury. These uncertainties underscore the difficulty of attributing individual organ manifestations in critically ill patients with disseminated viral infection.

In summary, this case demonstrates the fulminant potential of disseminated VZV infection in a child with ES and highlights the complexity of providing extracorporeal support in the setting of profound thrombocytopenia. Early recognition and antiviral therapy remain essential, whereas severe refractory cardiopulmonary failure may warrant consideration of VA‐ECMO even when bleeding risk is substantial. Individualized anticoagulation and aggressive platelet support may facilitate extracorporeal support in carefully selected patients with severe hematologic dysfunction.

## Funding

No funding was received for this manuscript.

## Consent

All the patients allowed personal data processing, and informed consent was obtained from all individual participants included in the study.

## Conflicts of Interest

The authors declare no conflicts of interest.

## Data Availability

The data that support the findings of this study are available on request from the corresponding author. The data are not publicly available due to privacy or ethical restrictions.
